# Thrombotic Thrombocytopenia Masquerading As COVID-19 Infection

**DOI:** 10.7759/cureus.26933

**Published:** 2022-07-17

**Authors:** Krishna Desai, Aarthi Sridhar, Jose Matos, Sana Mulla, Rajesh Thirumaran

**Affiliations:** 1 Internal Medicine, Mercy Catholic Medical Center, Philadelphia, USA; 2 Hematology/Oncology, Mercy Catholic Medical Center, Philadelphia, USA

**Keywords:** hemolysis, plasmapheresis, schistocytes, covid-19, thrombotic thrombocytopenic purpura

## Abstract

Thrombotic thrombocytopenic purpura (TTP) is a rare and challenging diagnosis that consists of thrombotic microangiopathy due to complete or severe deficiency of ADAMTS13 protease that can present at any age. It is very important to have a suspicion concerning this disease as mortality can be very high if it goes unnoticed. This case describes a patient that presented with gastrointestinal symptoms and hematuria and was found to have COVID-19 and TTP.

We present a case of a 40-year-old female with no past medical history who presented to the Emergency Department with complaints of abdominal pain, nausea, vomiting, and dark urine. The patient workup revealed a platelet count of 4000. The patient was also noted to be COVID-19 positive. Upon further workup, the TTP diagnosis was confirmed. She responded appropriately to plasmapheresis and steroids.

COVID-19 seems to be linked to a wide range of hematologic conditions including but not limited to TTP. In view that TTP can have significant mortality if untreated, we must be suspicious about this condition in COVID-19 cases. The aim of this case report is to highlight the importance of having a low threshold for making a diagnosis of TTP if labs are significant for hemolytic anemia. Our aim is also to emphasize that the treatment should be initiated if schistocytes are seen on the peripheral smear without awaiting laboratory results confirming low levels of ADAMTS13, given the fatal nature of the condition if left untreated.

## Introduction

Thrombotic thrombocytopenic purpura (TTP) is a rare thrombotic microangiopathy due to complete or severe deficiency of ADAMTS13 (A Disintegrin And Metalloprotease with a ThromboSpondin type 1 motif, member 13) protease. It can be hereditary, immune-mediated, or drug-induced. The multiple small vessel platelet thrombi systemically occlude the arterioles leading to ischemia and organ dysfunction by hyaline-induced occlusion. This sets the pentad of symptoms such as fever, microangiopathic hemolytic anemia (MAHA), thrombocytopenia, renal dysfunction, and neurologic abnormalities. It can be inherited or acquired. Only ~5% of cases are thought to be inherited. Most cases are acquired and present as a life-threatening medical emergency [1,2]. This case report is of 40-year-old female who presented with thrombocytopenia of 4000 and in the presence of an underlying COVID-19 infection, potentially linking COVID-19 as a trigger for immune-mediated TTP. 

## Case presentation

A 40-year-old African-American female with no past medical history presented to the hospital with complaints of vague abdominal pain and nausea for 1 day, followed by bloody vomiting, bloody bowel movements, and bloody urine for 1 day. The abdominal pain was radiating to the left flank, 7/10 in intensity, with no particular aggravating or relieving factors. She reported taking only 1 dose of COVID vaccine on 05/14/2021, i.e. 5 months 11 days before her presentation. Otherwise, her review of systems was negative. 

**Figure 1:**
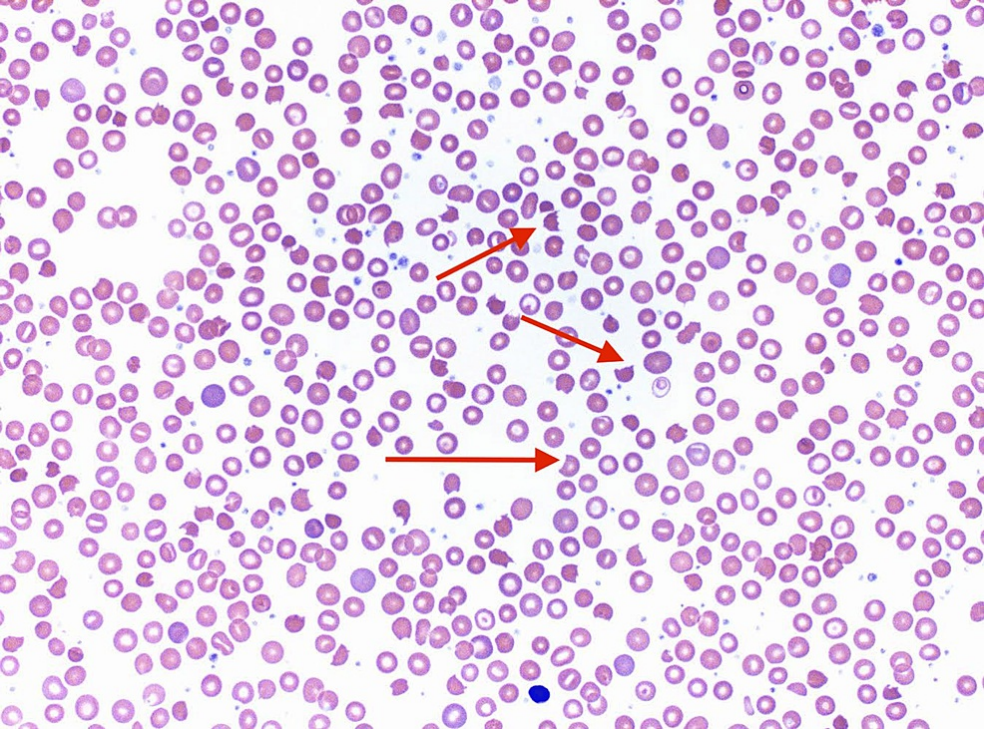
Peripheral smear of the patient showing schistocytes

Physical examination was remarkable for a temperature of 100.3, heart rate of 97 beats/min, tachypnea with 22 breaths/min, SpO2 99% RA, mild icterus, dry mucous membranes, epigastric tenderness, nonpalpable purpuric lesions of 3x3.3cm on the right thigh. Her labs (Table 1) were notable for a positive COVID test, low platelet count of 4000/UL, normocytic anemia with hemoglobin of 9.6 g/dL, mean corpuscular volume of 92, potassium of 2.9 mEq/L, lipase of 265 U/L, hyperbilirubinemia with a total bilirubin of 8.4 mg/dL, direct bilirubin of 0.5 mg/dl, elevated troponin of 310 ng/L, AST of 54 U/L, creatinine of 0.90, blood urea nitrogen of 26 mg/dL, and blood glucose of 117 mg/dL. The coagulation panel (prothrombin time, INR, and APTT) was within normal limits. Urine analysis showed urine RBC > 100. Given the high suspicion for TTP, she was started on high-dose steroids and the blood bank was contacted for plasma exchange. On Day 2, the lab findings showed a low haptoglobin <30, percent reticulocytes 2.7, ferritin 457, and lactate dehydrogenase of 2158. Peripheral smear was notable for multiple schistocytes (Figure 1), making TTP more likely. Coombs was negative. PLASMIC score on admission was 6, indicating a 72% risk of severe ADAMTS13 deficiency (defined as ADAMTS13 activity level <15%) [5]. ADAMTS-13 levels were sent.

**Table 1:** Significant Laboratory Tests

Labs	Lab value	On discharge
WBC	8.8 Thou/uL	12.6 Thou/uL
RBC	2.89 Mill/uL	2.59Mill/uL
Hemoglobin	9.6 gm/dL --> 6.8 gm/dL	8.9 gm/dL
Platelets	4 Thou/uL	170 Thou/uL
PT	14.3 seconds	
INR	1.2	
APTT	30.5 seconds	
Fibrinogen	472 g/L	
D-Dimer	12.86 ng/mL	
LDH	2158 units/L	
Creatinine	0.90	0.70
ANA	Negative	
Proteinase 3	<1.0	
Myeloperoxidase	<1.0	
SARS-CoV 2 (RT PCR)	Detected	
ADAMTS 13	<0.03 IU/mL	0.76 IU/mL

**Figure 2:**
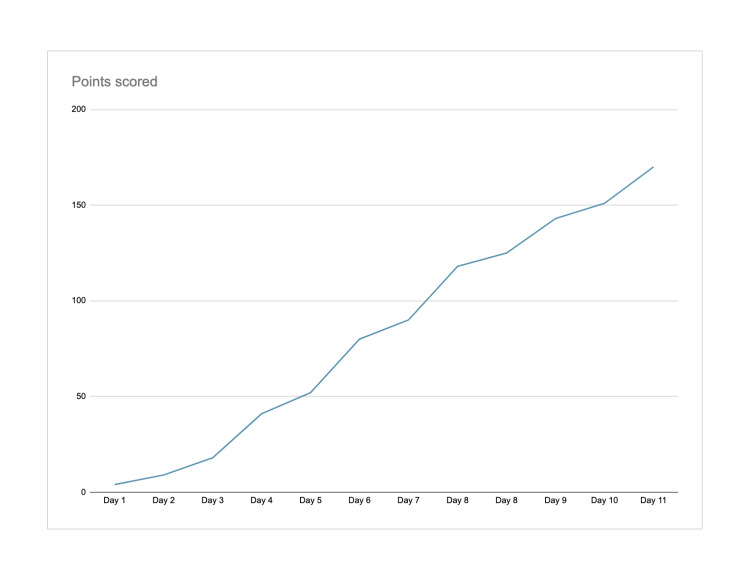
Platelet trend from an initial presentation on Day 1 to Day 11, gradually up-trending after initiating treatment.

In the interim, she received 1 unit of packed red blood cells (PRBC) transfusion. She was started on plasmapheresis on Day 1 of her admission and received a total of 2 sessions until her platelet count reached 150,000 (2 days later). Her platelets continued to trend upwards throughout her course of admission (Figure 2). Intravenous (IV) methylprednisolone 1,000mg daily was given in the first 3 days and was then transitioned to prednisone 1mg/kg. Initiation of Rituxan and Caplacizumab (a novel agent- that inhibits the vWF-platelet glycoprotein-Ib interaction, and blocks the adhesion of platelets to vWF multimers) were contemplated. However, they were not started due to active COVID-19 infection. The ADAMTS-13 levels was followed up over the next few days and was positive for critically low activity of <0.03. Levels of ADAMTS-13 rechecked on Day 11 of hospitalization showed an increase from <0.03 on Day 1 to 0.76 IU/mL. The patient was deemed medically stable for discharge with a platelet count of 170 Thou/uL. Prednisone 1mg/kg was tapered after ADAMTS 13 levels were >20% over 2-3 weeks post-discharge. 

She was followed up in the outpatient hematology clinic where her ADAMTS-13 was rechecked after 3 months which was at 1.01. She continued to remain on the prednisone taper and the patient mentioned that she was back to her baseline with no new bleeding episodes and complete resolution of her symptoms. Her ADAMTS-13 levels will be monitored every 3 months for 2 years and then annually.

## Discussion

The annual incidence is 1.5-6 cases per million per year in adults. Risk factors for TTP include the female gender, African American population (increased incidence likely due to decreased expression of HLA-DRB1*04 which is a protective allele) as well as obesity, of which, all were present in our case [1,2]. There have been an increasing number of cases of TTP being reported due to COVID-19.

ADAMTS13 is a metalloprotease primarily produced in the liver, but is also found in the endothelium and megakaryocytes. It cleaves the large polymers of VonWillebrand’s Factor (VWF). TTP is caused by severe ADAMTS-13 deficiency, usually <10% of normal. Reduced activity or complete deficiency leads to the accumulation of these large molecules causing platelets to accumulate, leading to thrombus formation which results in systemic ischemia, organ damage, and microangiopathies [3]. Despite the severe deficiency of ADAMTS-13, symptoms may not be clinically apparent until triggered. These triggers include autoimmune diseases, cancers, antineoplastic therapies, bone marrow and solid organ transplants, drugs, and pregnancy. Recent evidence suggests viral infections to be an established trigger in setting up an acute episode of TTP. The precise pathogenesis of viral-associated TTP is not clearly understood, however, cytokine storm-induced endothelial injury, immune complexes and in particular, COVID-19 induce an exaggerated production of ADAMTS-13 inhibitors. In addition to these, SARS-COV-2 infection causes a severe systemic inflammation which in turn causes diffuse activation of the microvascular endothelium setting acute onset of TTP [4]. The standard of care after several clinical trial results and real-world data has demonstrated the efficacy and safety of the triple therapy consisting of therapeutic plasma exchange, caplacizumab, and immunosuppressives (e.g., corticosteroids and rituximab) for acute TTP. Such a strategy has significantly accelerated the normalization of platelet counts, decreased the length of stays in the intensive care unit and the hospital, and most importantly reduced the mortality rate [5].

During the initial COVID pandemic, due to the deficiency of plasma exchange as well as in special conditions like- Jehovah’s witnesses (abstain from blood and blood product transfusions), there emerged alternate methods to treat TTP without the use of plasma exchange. There was a case reported about a patient diagnosed with a clinical relapse of acquired immune-mediated thrombotic thrombocytopenic purpura (TTP) who was successfully treated with low-dose rituximab plus corticosteroids. Rituximab 100 mg weekly for 4 weeks was administered, combined with 1 mg/kg of prednisone, obtaining a complete hematological response in 6 weeks. This case suggests that plasma exchange may be unnecessary for a subset of patients with relapsed TTP who are clinically stable without significant end-organ damage [6].

Immunomodulatory agents such as vincristine and cyclosporine were popular as salvage therapy for patients with a poor response to plasma exchange in the pre-rituximab era. Splenectomy has been used to treat patients with refractory and relapsing TTP as well as prophylactic therapy in patients with multiple relapses. Other novel therapies include the usage of N-acetyl cysteine, bortezomib, anfebatide, and usage of recombinant ADAMTS-13 [7,8].

## Conclusions

A high index of suspicion for TTP should be maintained, especially in the setting of COVID-19, in patients with markedly elevated lactate dehydrogenase, elevated d-dimer, severe thrombocytopenia, and presence of schistocytes on peripheral smear, especially since ADAMTS-13 levels in the blood may take longer to result. Given the nature of critical conditions which can be fatal, there should be no delay in the initiation of plasmapheresis such as with our patient who received plasmapheresis on Day 1 of her admission. 
